# Supplementary material for: Lack of protective effect of chloroquine derivatives on COVID-19 disease in a Spanish sample of chronically treated patients
Source: PLoS One. 2020 Dec 14;15(12):e0243598. doi: 10.1371/journal.pone.0243598 (PMC7735637; doi:10.1371/journal.pone.0243598)

**Lack of protective effect of chloroquine derivatives on COVID-19 disease in a Spanish sample of chronically treated patients.**

Marina Laplana, Oriol Yuguero, Joan Fibla

**S1 Text**. Survey questions in Spanish

* Obligatoria

Sección 1.- Datos demográficos

1. Edad *

***Marcar solo una opción***

18 a 30


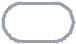


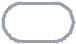
 31 a 50


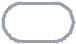
 51 a 65


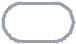
 mayor de 65

1. Sexo *

***Marcar solo una opción***


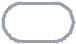
 Mujer


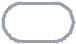
 Hombre

Prefiero no indicarlo


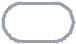


1. Lugar de residencia (Provincia) *

__________________________

| - - - Araba     - Albacete     - Alicante     - Almería     - Asturias     - Ávila     - Badajoz     - Barcelona     - Burgos     - Cáceres     - Cádiz     - Cantabria     - Castellón     - Ceuta     - Ciudad Real     - Córdoba     - Cuenca     - Girona | - - - Granada     - Guadalajara     - Guipúzcoa     - Huelva     - Huesca     - Illes Balears     - Jaén     - A Coruña     - La Rioja     - Las Palmas     - León     - Lleida     - Lugo     - Madrid     - Málaga     - Melilla     - Murcia     - Navarra | - - - Ourense     - Palencia     - Pontevedra     - Salamanca     - Santa Cruz de Tenerife     - Segovia     - Sevilla     - Soria     - Tarragona     - Teruel     - Toledo     - València     - Valladolid     - Bizkaia     - Zamora     - Zaragoza     - Fuera del territorio español |
| --- | --- | --- |

1. ¿Está usted tomando regularmente cloroquina o alguno de sus derivados? (marcas comerciales: Aralén HCl, Axemal, Dolquine, Ilinol, Quensyl , Plaquenil, Resochín) *

***Marcar solo una opción***


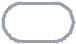
 Sí


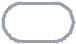
 No


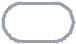
 No, solo lo he tomado de forma esporádica como antimalárico o para el tratamiento de una infección

Responda sólo en caso de respuesta afirmativa a la pregunta anterior

En caso contrario pase a la siguiente sección

1. Indique la marca del medicamento que está usted tomando

***Marqueu només un oval per fila.***


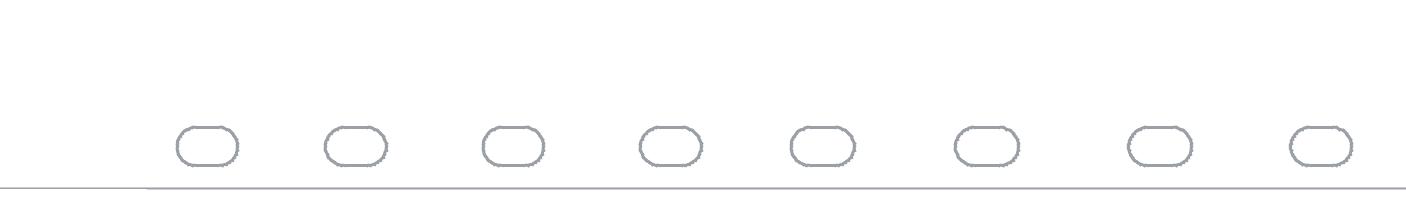


| Aralén | Axemal Dolquine | Ilinol | Quensyl Plaquenil Resochín | Otro |  |
| --- | --- | --- | --- | --- | --- |
| HCl |  |  |  |  |  |
|  |  |  |  |  |  |
|  |  |  |  |  |  |

**F**il**a 1**

1. ¿Desde cuándo está usted tomando este medicamento?

***Marcar solo una opción***


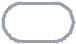
 Menos de tres meses


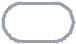
 Más de tres meses

Sección 2 .- Datos sobre su salud

1. ¿Ha sido usted diagnosticado/a positivo para COVID19? *

***Marcar solo una opción***


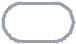
 Sí


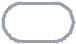
 No

1. En caso de respuesta afirmativa a la pregunta anterior ¿Precisó hospitalización para el tratamiento de la infección?

***Marcar solo una opción***


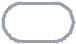
 Sí, seguimiento hospitalario sin complicaciones


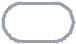
 Sí, seguimiento hospitalario con cuidados intensivos


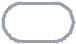
 No, el seguimiento ha sido domiciliario

1. Si usted NO ha sido diagnosticado positivo para COVID19: ¿En los últimos tres meses ha presentado uno o más de uno de los siguientes síntomas?

***Seleccioneu totes les opcions que corresponguin.***


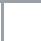

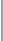
 Tos seca, continua y persistente


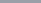


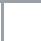

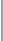
 Dolor de garganta


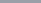


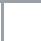

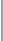
 Dificultad para respirar


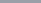


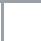

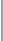
 Pérdida del gusto y/o olfato


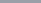


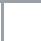

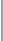
 Fiebre


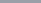


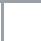

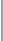
 Malestar general


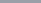


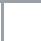

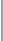
 Mareo i/o vómitos


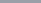


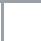

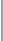
 No he tenido ninguno de estos síntomas


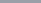


1. En caso de haber manifestado alguno de los síntomas anteriores

***Marcar solo una opción***


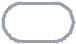
 Los síntomas han durado MENOS de tres días y NO he tenido que ir al médico


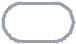
 Los síntomas han durado MÁS de tres días pero NO he tenido que ir al médico
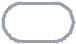
 Los síntomas han durado MÁS de tres días y SÍ que he tenido que ir al médico

Sección 3.- Datos sobre el grado de exposición

1. ¿En los últimos tres meses ha participado en alguna actividad donde haya habido una alta concentración de gente?

***Marcar solo una opción***


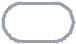
 Sí


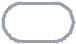
 No


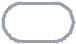
 No lo recuerdo

1. En caso de respuesta afirmativa a la pregunta anterior, indique cual ha sido esa actividad.


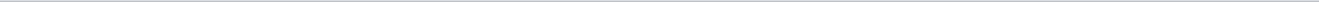

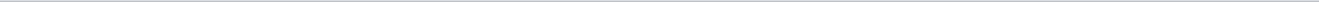

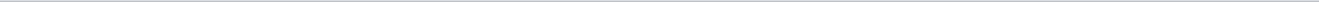

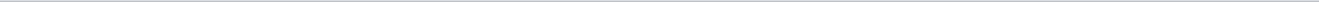

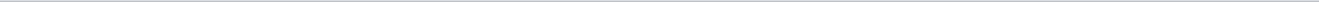


1. ¿Alguna persona de su entorno de proximidad con quien haya estado en contacto ha sido diagnosticado/a positivo para COVID19?

***Marcar solo una opción***


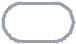
 Sí


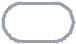
 No


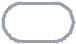
 No lo sé

1. En caso de respuesta afirmativa a la pregunta anterior ¿Qué relación tiene con usted esta persona?

***Marcar solo una opción***


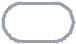
 Familiar con quien convivo


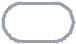
 Amigo/a conocido/a con quien ha estado en contacto cercano


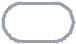
 Amigo/a conocido/a con quien ha estado en contacto indirecto

1. ¿Alguna persona de su entorno de proximidad con quien haya estado en contacto en los últimos tres meses ha presentado uno o más de uno de los siguientes síntomas?

***Seleccioneu totes les opcions que corresponguin.***


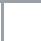

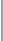
 Tos seca, continuada y persistente


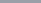


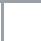

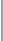
 Dolor de garganta


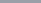


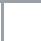

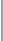
 Dificultad para respirar


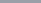


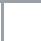

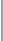
 Pérdida del gusto y / o olfato


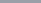


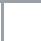

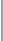
 Fiebre


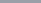


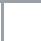

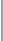
 Malestar general


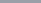


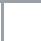

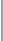
 Mareo y/o vómitos


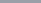


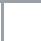

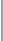
 Ninguna persona cercana a mi ha tenido estos síntomas


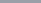


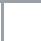

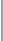
 No lo se


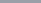


1. En caso de que alguien próximo a usted haya manifestado alguno de los síntomas anteriores, ¿Qué relación tiene con usted esta persona?

***Marcar solo una opción***


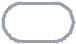
 Familiar con quien convivo


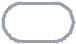
 Amigo/a conocido/a con quien ha estado en contacto cercano


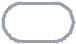
 Amigo/a conocido/a con quien ha estado en contacto indirecto

1. Si lo desea puede añadir un comentario u observación que considere relevante para el estudio.


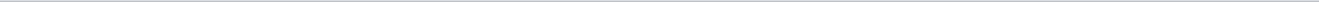

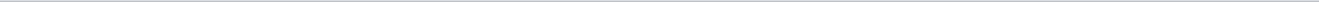

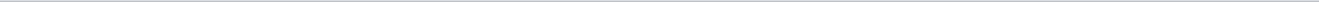

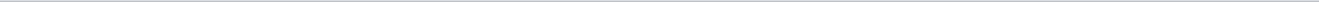

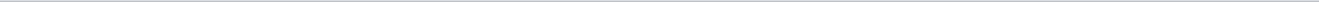

Supplement: S1 Text — (DOCX) [file pone.0243598.s001.docx]
