# Supplementary material for: Lack of protective effect of chloroquine derivatives on COVID-19 disease in a Spanish sample of chronically treated patients
Source: PLoS One. 2020 Dec 14;15(12):e0243598. doi: 10.1371/journal.pone.0243598 (PMC7735637; doi:10.1371/journal.pone.0243598)

**Lack of protective effect of chloroquine derivatives on COVID-19 disease in a Spanish sample of chronically treated patients.**

Marina Laplana, Oriol Yuguero, Joan Fibla

**S2 Text.** Survey questions in Catalan

* Obligatòria

Secció 1.- Dades demogràfiques

1. Edat *

***Marqueu només una opció.***

18 a 30


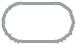


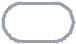
 31 a 50


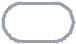
 51 a 65


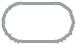
 major de 65

1. Sexe *

***Marqueu només una opció.***


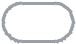
 Dona


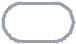
 Home


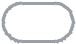
 Prefereixo no indicar-ho

1. Lloc de residencia (Província) *

| - - - Araba     - Albacete     - Alicante     - Almería     - Asturias     - Ávila     - Badajoz     - Barcelona     - Burgos     - Cáceres     - Cádiz     - Cantabria     - Castellón     - Ceuta     - Ciudad Real     - Córdoba     - Cuenca     - Girona | - - - Granada     - Guadalajara     - Guipúzcoa     - Huelva     - Huesca     - Illes Balears     - Jaén     - A Coruña     - La Rioja     - Las Palmas     - León     - Lleida     - Lugo     - Madrid     - Málaga     - Melilla     - Murcia     - Navarra | - - - Ourense     - Palencia     - Pontevedra     - Salamanca     - Santa Cruz de Tenerife     - Segovia     - Sevilla     - Soria     - Tarragona     - Teruel     - Toledo     - València     - Valladolid     - Bizkaia     - Zamora     - Zaragoza     - Fuera del territorio español |
| --- | --- | --- |

1. Està vostè prenent regularment cloroquina o algun dels seus derivats? (Marques comercials: Aralén HCl, Axemal, Dolquine, Ilinol, Quensyl, Plaquenil, Resochin). *

***Marqueu només una opció.***


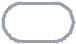
 Sí


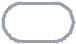
 No


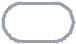
 No, només l'he pres de manera esporàdica com antimalàric o pel tractament d'una infecció

Respongui només en cas de resposta afirmativa a la pregunta anterior

En cas contrari passi a la següent secció del qüestionari

1. Indiqueu la marca del medicament que està vostè prenent.

***Marqueu només un oval per fila.***


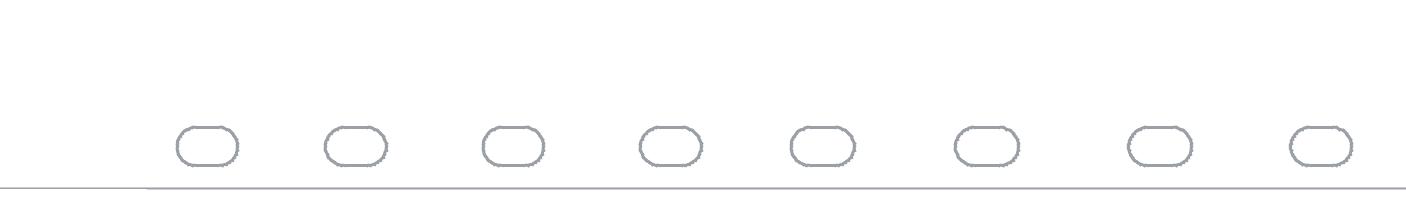


| Aralén | Axemal Dolquine | Ilinol | Quensyl Plaquenil Resochín | Altre |  |
| --- | --- | --- | --- | --- | --- |
| HCl |  |  |  |  |  |
|  |  |  |  |  |  |
|  |  |  |  |  |  |

**F**il**a 1**

1. Des de quan està vostè prenent aquest medicament?

***Marqueu només una opció.***


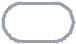
 Menys de tres mesos


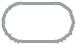
 Més de tres mesos

Secció 2.- Dades sobre la seva salut

1. Ha estat vostè diagnosticat/da positiu per COVID19? *

***Marqueu només una opció.***


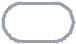
 Sí


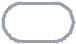
 No

1. En cas de resposta AFIRMATIVA a la pregunta anterior ¿Ha precisat hospitalització pel tractament de la infecció?

***Marqueu només una opció.***


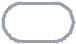
 No, el seguiment ha estat domiciliari


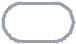
 Sí, seguiment hospitalari sense complicacions


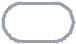
 Sí, seguiment hospitalari amb cures intensives

1. Si vostè NO ha estat diagnosticat positiu per COVID19: ¿En els últims tres mesos ha presentat un o més d'un dels següents símptomes?

***Seleccioneu totes les opcions que corresponguin.***


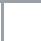

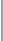
 Tos seca, continuada i persistent


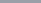


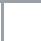

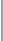
 Mal de coll


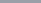


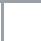

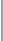
 Dificultat per respirar


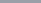


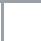

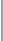
 Pèrdua del gust i/o olfacte


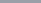


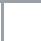

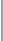
 Febre


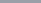


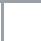

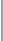
 Malestar general


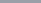


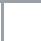

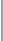
 Mareig i/o vòmits


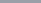


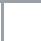

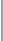
 No he tingut cap d'aquests símptomes


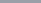


1. En cas d'haver manifestat algun dels símptomes anteriors.

***Marqueu només una opció.***


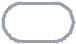
 Els símptomes han durat MENYS de tres dies i NO he hagut d'anar a el metge


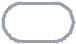
 Els símptomes han durat MÉS de tres dies però NO he hagut d'anar a el metge
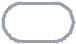
 Els símptomes han durat MÉS de tres dies i SÍ que he hagut d'anar a el metge

Secció 3.- Dades sobre el grau d'exposició

1. En els últims tres mesos ha participat en alguna activitat on hi hagi hagut una alta concentració de gent?

***Marqueu només una opció.***


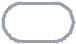
 Sí


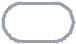
 No


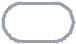
 No ho recordo

1. En cas de resposta AFIRMATIVA a la pregunta anterior, indiqui quina ha estat aquesta activitat.


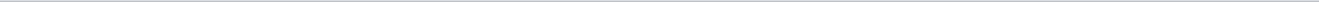

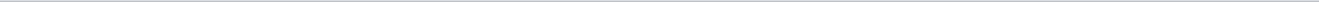

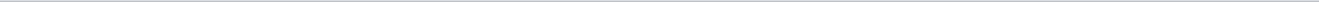

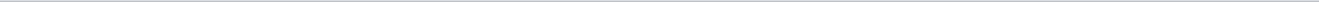

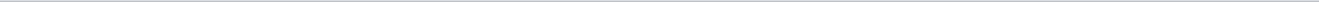


1. Alguna persona del seu entorn de proximitat amb qui hagi estat en contacte ha estat diagnosticat/da POSITIU per COVID19?

***Marqueu només una opció.***


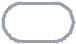
 Sí


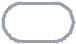
 No


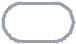
 No ho sé

1. En cas de resposta AFIRMATIVA a la pregunta anterior ¿Quina relació té amb vostè aquesta persona?

***Marqueu només una opció.***


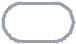
 Familiar amb qui convisc


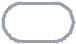
 Amic/ga conegut/da amb qui he estat en contacte proper


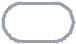
 Amic/ga conegut/da amb qui he estat en contacte indirecte

1. Alguna persona del seu entorn de proximitat amb qui hagi estat en contacte en els últims tres mesos ha presentat un o més d'un dels següents símptomes?

***Seleccioneu totes les opcions que corresponguin.***


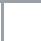

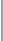
 Tos seca, continuada i persistent


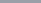


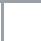

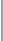
 Mal de coll


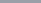


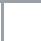

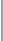
 Dificultat per respirar


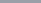


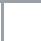

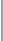
 Pèrdua de el gust i/o olfacte


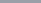


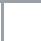

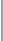
 Febre


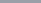


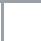

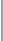
 Malestar general


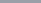


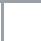

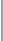
 Mareig i/o vòmits


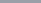


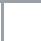

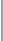
 Cap persona del meu entorn ha manifestat aquests símptomes


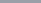


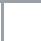

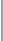
 No ho sé


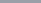


1. En el cas que alguna persona pròxima a vostè hagi manifestat aquests símptomes ¿Quina relació té amb vostè aquesta persona?

***Marqueu només una opció.***


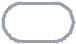
 Familiar amb qui convisc


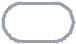
 Amic/ga conegut/da amb qui he estat en contacte proper


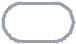
 Amic/ga conegut/da amb qui he estat en contacte indirecte

1. Si ho desitja pot afegir un comentari o observació que consideri rellevant per a l'estudi.


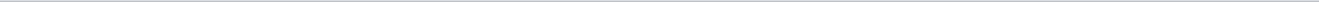

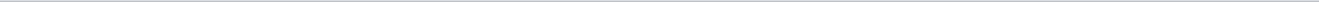

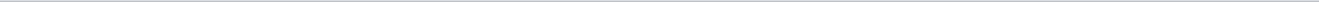

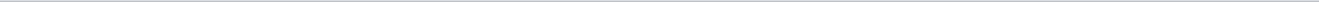

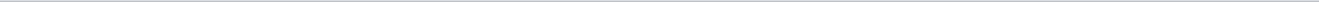

Supplement: S2 Text — (DOCX) [file pone.0243598.s002.docx]
