# Supplementary material for: Lack of protective effect of chloroquine derivatives on COVID-19 disease in a Spanish sample of chronically treated patients
Source: PLoS One. 2020 Dec 14;15(12):e0243598. doi: 10.1371/journal.pone.0243598 (PMC7735637; doi:10.1371/journal.pone.0243598)
Supplement: S3 Text — (DOCX) [file pone.0243598.s003.docx]

**Lack of protective effect of chloroquine derivatives on COVID-19 disease in a Spanish sample of chronically treated patients.**

Marina Laplana, Oriol Yuguero, Joan Fibla

**S3 Text.** Survey questions translated to English

* Mandatory

Section 1.- Demographic data

1. Age *

***Check only one option***

18 to 30


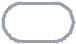


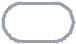
 31 to 50


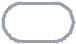
 51 to 65


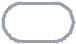
 over 65

1. Sex *

***Check only one option***


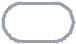
 Woman


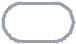
 Man


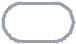
 I prefer not to indicate it

1. Place of residence (Province) *

______________________________

| - - - Araba     - Albacete     - Alicante     - Almería     - Asturias     - Ávila     - Badajoz     - Barcelona     - Burgos     - Cáceres     - Cádiz     - Cantabria     - Castellón     - Ceuta     - Ciudad Real     - Córdoba     - Cuenca     - Girona | - - - Granada     - Guadalajara     - Guipúzcoa     - Huelva     - Huesca     - Illes Balears     - Jaén     - A Coruña     - La Rioja     - Las Palmas     - León     - Lleida     - Lugo     - Madrid     - Málaga     - Melilla     - Murcia     - Navarra | - - - Ourense     - Palencia     - Pontevedra     - Salamanca     - Santa Cruz de Tenerife     - Segovia     - Sevilla     - Soria     - Tarragona     - Teruel     - Toledo     - València     - Valladolid     - Bizkaia     - Zamora     - Zaragoza     - Fuera del territorio español |
| --- | --- | --- |

1. Are you regularly taking chloroquine or any of its derivatives? (trademarks: Aralén HCl, Axemal, Dolquine, Ilinol, Quensyl, Plaquenil, Resochín)*

***Check only one option***


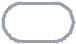
 Yes


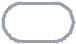
 Not


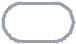
 No, I have only taken it sporadically as an antimalarial or to treat an infection

Answer only in case of affirmative answer to the previous question

Otherwise go to next section

1. Indicate the brand of medicine you are taking

***Mark only one oval per row.***


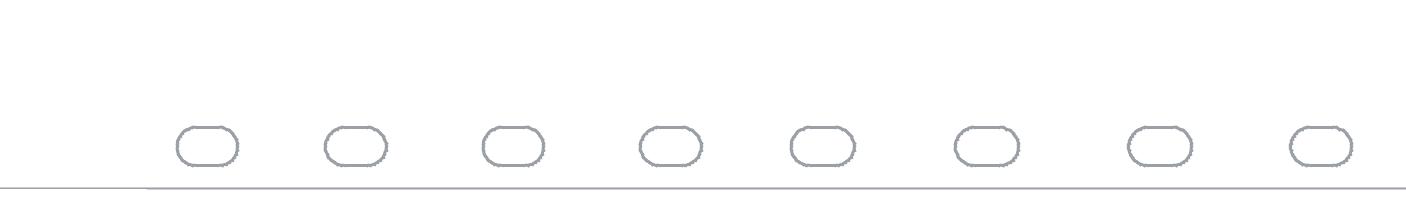


| Aralén | Axemal Dolquine | Ilinol | Quensyl Plaquenil Resochín | Other |  |
| --- | --- | --- | --- | --- | --- |
| HCl |  |  |  |  |  |
|  |  |  |  |  |  |
|  |  |  |  |  |  |

**F**ila 1

1. How long have you been taking this medicine?

***Check only one option***


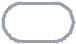
 Less than three months


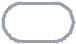
 More than three months

Section 2.- Facts about your health

1. Have you been diagnosed positive for COVID-19? *

***Check only one option***


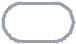
 Yes


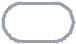
 Not

1. If the answer to the previous question was affirmative, did you require hospitalization to treat the infection?

***Check only one option***


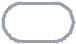
 Yes, hospital follow-up without complications


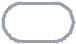
 Yes, hospital follow-up with intensive care


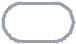
 No, the follow-up has been at home

1. If you have NOT been diagnosed positive for COVID-19: In the last three months have you had one or more of the following symptoms?

***Select all your corresponding options.***


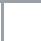

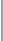
 Dry, continuous and persistent cough


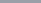


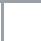

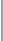
 Sore throat


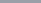


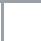

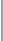
 Difficulty breathing


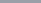


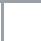

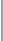
 Loss of taste and / or smell


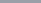


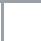

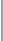
 Fever


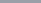


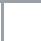

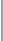
 General discomfort


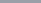


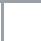

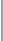
 Dizziness and / or vomiting


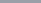


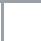

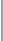
 I have not had any of these symptoms


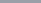


1. In case of having manifested any of the above symptoms

***Check only one option***


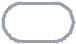
 The symptoms have lasted LESS than three days and I have NOT had to go to the doctor

The symptoms have lasted MORE than three days but I have NOT had to go to the doctor


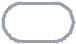


Symptoms have lasted MORE than three days and I HAVE had to go to the doctor


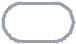


Section 3.- Data on the degree of exposure

1. In the last three months, have you participated in any activity where there has been a high concentration of people?

***Check only one option***


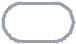
 Yes


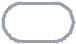
 Not


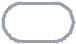
 I do not remember

1. In case of affirmative answer to the previous question, indicate what that activity has been.
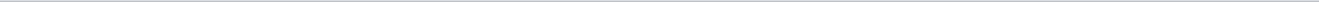

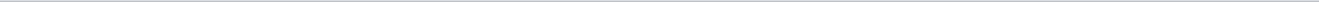

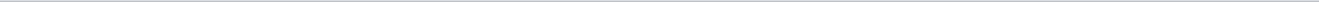

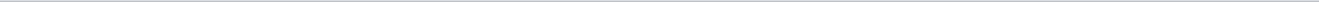

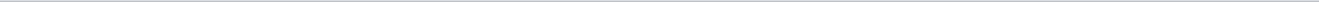

2. Has anyone in your immediate environment with whom you have been in contact been diagnosed positive for COVID-19?

***Check only one option***


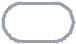
 Yes


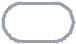
 Not


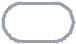
 I do not know

1. In case of affirmative answer to the previous question, what relationship does this person have with you?

***Check only one option***

- Family member with whom I live
- Friend / acquaintance with whom I have been in close contact
- Friend / acquaintance with whom I have been in indirect contact

1. Has anyone in your immediate environment with whom you have been in contact in the last three months have one or more of the following symptoms?

***Select all your corresponding options.***


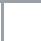

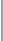
 Dry, ongoing and persistent cough


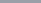


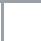

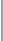
 Sore throat


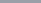


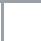

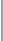
 Difficulty breathing


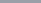


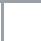

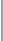
 Loss of taste and / or smell


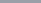


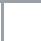

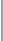
 Fever


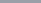


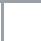

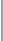
 General discomfort


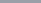


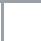

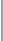
 Dizziness and / or vomiting


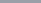


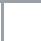

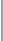
 No person close to me has had these symptoms


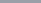


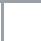

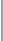
 I do not know


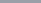


1. In case someone close to you has manifested any of the above symptoms, what relationship does this person have with you?

***Check only one option***


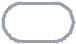
 Family member with whom I live


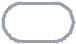
 Friend / acquaintance with whom I have been in close contact


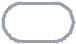
 Friend / acquaintance with whom I have been in indirect contact

1. If you wish, you can add a comment or observation that you consider relevant to the study.
