# Supplementary material for: Lack of protective effect of chloroquine derivatives on COVID-19 disease in a Spanish sample of chronically treated patients
Source: PLoS One. 2020 Dec 14;15(12):e0243598. doi: 10.1371/journal.pone.0243598 (PMC7735637; doi:10.1371/journal.pone.0243598)
Supplement: Attachment — Submitted filename: Response to Reviewers.docx [file pone.0243598.s005.docx]

18^th^, September 2020

Response to Academic Editor,

Dear Dr. Tan,

We greatly appreciate your comments and suggestions in relation to our submitted manuscript “Lack of protective effect of chloroquine derivatives on COVID-19 disease in a Spanish sample of chronically treated patients” (PONE-D-20-26952).

As you point out, we address one of the most urgent and heavily debated questions regarding intervention in the COVID-19 pandemic, namely (i.e.) whether or not chloroquine is effective on COVID-19. This question arose during the first month of the pandemic in response to controversial publications supporting a role of chloroquine derivatives, in special hydroxychloroquine, in protecting against COVID-19 disease. In fact, the Food and Drug Administration (FDA), via its Emergency Use Authorization (EUA) authority, on March 28^th^ provided authorization for use of hydroxychloroquine for treating patients with COVID-19, and it was not until June that this authorization was revoked. It was in this controversial context in which, at the end of March, we designed our survey in order to address whether chloroquine is effective against COVID-19.

In order to do so, we took advantage of the only available source of people receiving this drug chronically as a treatment, the group of patients with autoimmune diseases. Thus, our main objective was to know if the prevalence of declared COVID-19 disease in this group was different from that in people that do not receive this drug.

As important as the selection criteria were for inclusion in the treated group, also were the selection criteria for inclusion in the reference group. At the moment of designing the survey, in terms of the risk of infection, what had been observed was that a very low percentage of COVID-19 patients had an autoimmune disease (less than 1%) (Emmi, G., Bettiol, et al. 2020, Autoimmunity Reviews 19(7), 102575. https://dx.doi.org/10.1016/j.autrev.2020.102575), while age, gender and region of origin were the main known factors affecting SARS-CoV-2 infection in Spain's autonomies. According to this, we considered age, gender and region of origin as the main selection criteria for the untreated control group.

In addition, the set of untreated cases had to comprise a large number of individuals in order to be able to do the proper matching between treated and untreated individuals, with the treated patients being the limiting group. This matching process would have been difficult to achieve, in a reasonable time, if our control group came only from autoimmune disease patients without treatment.

As we considered that it was a priority to arrive at an answer as quickly as possible, and taking all the available evidence into account, we chose as reference group people from the general population, in order to be able to obtain a representative matchable group that would allow us to answer our question with the maximum guarantees of reliability.

We are aware that this design has limitations. Despite the lack of evidence that autoimmune diseases affect the risk of infection, we cannot completely discard a possible bias caused by the autoimmune status of cases. However, we assume a negligible effect caused by this possible bias in comparison with the other matching parameters such as sex, age and place of residence, that have allowed us to obtain a large and representative N of the different population groups. For these reasons, we consider that the selection of the general population as a reference group is appropriate and allows us to answer, with enough rigor, the main question of this project.

We have included some of these reflections in the justification of the inclusion criteria, as well as in the discussion section of the manuscript that we hope will allow readers to understand more precisely the scope and limitations of the work here presented.

Moreover, we have included a copy of the survey in Spanish, Catalan and an English translation and the minimal anonymized data set necessary to ensure replicability of our findings as Supporting Information. Furthermore, we confirm that the survey was validated and checked for inconsistencies.

Taking into account all the aforementioned considerations, we kindly ask you to consider this amended version of the manuscript for publication in Plos One.

Yours Sincerely,

Dr. Joan Fibla

Departament de Ciències Mèdiques Bàsiques

Universitat de Lleida-IRBLleida

Edifici Biomedicina I

Av. Rovira Roure, 80

25198 Lleida, Catalonia, Spain
